# 

**Published:** 1900-10-20

**Authors:** 


### NOTICE TO CORRESPONDENTS.

All MSS., Letters, Books for Review, and other matters intended for the Editor should be addressed The Editor, "The Hospital," The Hospital Building, 28 & 29 Southampton Street, Strand, W.C.

The Editor cannot undertake to return rejected MSS., even when accompanied by stamped directed envelope.

**Notice to Advertisers and Subscribers.**

All Advertisements, Orders for Copies of The Hospital, and Business Communications should be addressed to **THE MANAGER (Not to the Editor),** The Hospital, The Hospital Building, 28 & 29 Southampton Street, Strand, London, W.C.

The Cost of Subscription, post free, is as follows:—Per week, 2½d.; per per quarter, 2s. 8d.; per half-year, 5s. 3d.; per annum, 10s. 6d. Foreign.—Per annum, 15s. 2d., payable, in all cases, in advance.

**NOTICE.—Vol. XXVIII. of "The Hospital" (April to September, 1900), in handsome cloth binding, will shortly be on sale at the Office of this Journal, price, 7s. 6d. Cases for binding the Numbers contained in this Volume 1s. 6d. Index to Vol. XXVIII., 2½d., post free.**

**The Monthly Part for October is now ready. Price 6d., by post 8d.**

### BOOKS RECEIVED.

Baillière, Tindall and Cox.

"A Manual of Surgery for Students and Practitioners." By William Rose, M.B., B.S. Lond., F.R.C.S., and Albert Carless, M.S. Lond., F.R.C.S. Third Edition.

"Manual of Physiology, with Practical Exercises." By G. N. Stewart, M.A., D.Sc., M.D. Edin., D.P.H. Camb. Fourth Edition.

"A Synopsis of the British Pharmacopœia, 1898." By H. Whippell Gadd, with Analytical Notes by C. G. Moor, M.A. Cantab., F.I.C. Fifth Edition.

Smith, Elder and Co.

"Orthopædic Surgery: a Handbook." By Charles Bell Keetley, F.R.C.S.

Longmans, Green and Co.

"Malaria, according to the New Researches." By Professor Angelo Celli. Translated from the second Italian edition by John Joseph Eyre, M.R.C.P., L.R.C.S. Ire., D.P.H. Cambridge. With an Introduction by Dr. Patrick Manson.

William Blackwood and Sons.

"Twice Captured, a Record of Adventure during the Boer War." By the Earl of Rosslyn.

"How We Escaped from Pretoria." By Captain Aylmer Haldane, D.S.O., 2nd Batt. Gordon Highlanders.

H. K. Lewis.

"Illustrated Lectures on Nursing and Hygiene." By R. Lawton Roberts, M.D. Lond., D.P.H. Camb. Third Edition.

"The Student's Medical Dictionary." By George M. Gould, A.M., M.D. With a new table of Eponymic, Terms, and Tests.

John Bale, Sons, and Danielsson.

"Remarks on Colour Blindness and the Tests for Its Detection." By F. W. Edridge-Green, M.D., F.R.C.S.

Ville de Buénos-Ayres.

"Annuaire Statistique de la Ville de Buénos-Ayres."

J. W. Ward, Croydon.

"Where Should Londoners Live."

William Brown and Co.

"The Children's Home, Much Hadham, Herts."

### The Student of Medicine.

### APPOINTMENTS.

J. Percival Brown, M.B., Ch.B.Vict. Univ., appointed Junior House Surgeon to the Macclesfield General Infirmary. Charles E. Durrant, L.R.C.P.Lond., M.R.C.S.Eng., appointed House Physician to the General Lying-in Hospital, York Road, London. Charles Fisher, M.R.C.S., L.R.C.P., appointed Resident Medical Officer to the Victoria Hospital for Sick Children, Chelsea. Samson Gemmell, M.D.Glasg., appointed Professor of Clinical Medicine in the University of Glasgow. William Gillibrand, M.R.C.S., appointed Medical Referee under the Workmen's Compensation Act for Bolton, in County Court Circuit No. 5. K. C. Gimson, M.B., B.C.Camb., appointed Certifying Factory Surgeon for the Witham District of Essex. J. Basil Hall, M.C.Cantab., appointed Honorary Assistant Surgeon to the Bradford Royal Infirmary. Langworthy Laurie, M.B., Ch.B.Edin., appointed House Surgeon to the Victoria Hospital, Bournemouth. F. N. Menzies, M.B., Ch.B.Edin., appointed Senior Resident Physician at the Brompton Hospital for Consumption and Diseases of the Chest. W. J. Milligan, M.A., M.B., C.M., F.R.C.S. Edin., appointed Anæsthetist to the Great Northern Central Hospital. P. H. Mules, M.D.Edin., M.R.C.S.Eng., appointed Ophthalmic Surgeon to the Wrexham Infirmary. Arthur George Grant Plumley, M.B.Lond., M.R.C.S., L.R.C.P., L.D.S.Eng., appointed Dental Surgeon to the Clayton Hospital and Wakefield General Dispensary. H. H. Rubra, M.D.Brux., M.R.C.S. Eng., L.R.C.P.Lond., appointed Assistant Anæsthetist to the Great Northern Central Hospital. B. H. Slater, B.A., M.B.Camb., M.R.C.S., L.R.C.P., appointed Honorary Assistant Surgeon to the Bradford Royal Infirmary. F. Emslie Smith, M.B., C.M., appointed Junior House Surgeon to the Great Northern Central Hospital. Arthur D. Spence, M.B., Ch.B. Edin., appointed House Surgeon to Tottenham Hospital.

### Scraps and Gleanings.

The total amount collected at Peterborough this year on Hospital Saturday was £54 odd.

The Local Government Board have given their formal sanction to the borrowing by the Aldershot Council of £5,509 for hospital purposes. They however request that in future the Council would not carry out works, the cost of which it might be proposed to defray out of borrowed money, until the Board's sanction to the loan had been obtained.

In addition to the out-patients' waiting-room, the new wing of the Tunbridge Wells Eye and Ear Hospital will contain a laundry in the basement, and on the first floor an operating theatre with lantern light in the ceiling, cataract ward and bathrooms, and an external iron staircase will also be provided. Extensive alterations and improvements are also being made to the existing buildings.

The amount collected this year in connection with the Hungerford and District Hospital collections was £97 odd.

The Lord Mayor of Cork has received a cheque for £95 from the employees of the Cork Steamship Company in connection with the Hospital Saturday collection.

The motion that the proposed Torquay site should be purchased by the Newton Abbot Board of Guardians to provide cottage homes for the workhouse children was, at a recent meeting, rejected by 31 votes to 23. The seconder of the motion said he would bring the matter to the notice of the Local Government Board.

